# Supplementary material for: Cloud BioLinux: pre-configured and on-demand bioinformatics computing for the genomics community
Source: BMC Bioinformatics. 2012 Mar 19;13:42. doi: 10.1186/1471-2105-13-42 (PMC3372431; doi:10.1186/1471-2105-13-42)
Supplement: Additional file 1 — Supplementary 1 Cloud BioLinux software documentation in the form of a mini, self-contained website. Users need to download and uncompress the .zip file, and open through a web browser the "index.html" file available on the main directory. (ZIP 1823 kb). [file 1471-2105-13-42-S1.ZIP › Cloud-BioLinux-Package-Documentation/docs/ESTFerret.html]

Bio-Linux Software Documentation Pages

Back to search form

## ESTFerret

|  |  |
| --- | --- |
| Name | ESTFerret |
| Description | **ESTFerret** is a user-configurable, fully integrated pipeline for processing, clustering and annotating EST sequence data.  Sequence traces input to the system are read using phred and vector clipped and cleaned using lucy. A file suitable for downstream processing as well as a file ready for submission to the dbEST database are produced.  Clustering is done using cap3 twice with different parameters.  Annotation includes blasting against databases defined by the user and parsing these results, a pfscan search of the Prosite database, running GoMiner for annotating with GO categories, and ECprofiler.  Results are stored in a series of tab-delimited text tables. |
| Homepage |  |
| Remote Documentation |  |
